# Linkages between human health and ocean health: a participatory climate change vulnerability assessment for marine mammal harvesters

**DOI:** 10.3402/ijch.v72i0.20715

**Published:** 2013-08-05

**Authors:** Lily Gadamus

**Affiliations:** Natural Resources Division, Kawerak, Inc., Nome, Alaska, USA

**Keywords:** qualitative methods, climate change, adaptation, vulnerability, food security, indigenous

## Abstract

**Background:**

Indigenous residents of Alaska's Bering Strait Region depend, both culturally and nutritionally, on ice seal and walrus harvests. Currently, climate change and resultant increases in marine industrial development threaten these species and the cultures that depend on them.

**Objective:**

To document: (a) local descriptions of the importance of marine mammal hunting; (b) traditional methods for determining if harvested marine mammals are safe to consume; and (c) marine mammal outcomes that would have adverse effects on community health, the perceived causes of these outcomes, strategies for preventing these outcomes and community adaptations to outcomes that cannot be mitigated.

**Design:**

Semi-structured interviews and focus groups were conducted with 82 indigenous hunters and elders from the Bering Strait region. Standard qualitative analysis was conducted on interview transcripts, which were coded for both inductive and deductive codes. Responses describing marine mammal food safety and importance are presented using inductively generated categories. Responses describing negative marine mammal outcomes are presented in a vulnerability framework, which links human health outcomes to marine conditions.

**Results:**

Project participants perceived that shipping noise and pollution, as well as marine mammal food source depletion by industrial fishing, posed the greatest threats to marine mammal hunting traditions. Proposed adaptations primarily fell into 2 categories: (a) greater tribal influence over marine policy; and (b) documentation of traditional knowledge for local use. This paper presents 1 example of documenting traditional knowledge as an adaptation strategy: traditional methods for determining if marine mammal food is safe to eat.

**Conclusions:**

Participant recommendations indicate that 1 strategy to promote rural Alaskan adaptation to climate change is to better incorporate local knowledge and values into decision-making processes. Participant interest in documenting traditional knowledge for local use also indicates that funding agencies could support climate change adaptation by awarding more grants for tribal research that advances local, rather than academic, use of traditional knowledge.

The Bering Strait Region is facing both rapid climactic changes and accelerating industrial development (1), which affect the marine environment that provides both food (an estimated 2688.5 lbs per household annually) and cultural identity to Inupiat, Yup'ik and St. Lawrence Island Yupik residents (2). Marine mammal hunters in this region have observed considerable changes in ice extent, quality and behavior, which have also been documented in the scientific literature (3–6). As ice seals and walruses use sea ice for pupping and calving, resting, feeding and transportation (7, 8), concern about the future of these species has triggered status reviews under the Endangered Species Act for walruses and all 4 species of ice seals. Additionally, sea ice reduction means that marine traffic through the Bering Strait, as well as Arctic resource development, will most likely increase dramatically (1). Finally, the North Pacific Fisheries Management Council has commissioned a draft research plan for the Northern Bering Sea, and the National Marine Fisheries Service conducted a research trawl in 2010, both of which are pre-cursors to a possible opening of the area to bottom trawl fishing (9).

## Vulnerability analyses

As defined by Turner et al. (10, p. 8074), “Vulnerability is the degree to which a system, subsystem, or system component is likely to experience harm due to exposure to a hazard, either a perturbation or a stress/stressor”. Vulnerability analyses provide policymakers with the information needed for decisions that preserve human and environmental well-being in the face of stressors such as climate change (10). As attempts to mitigate climate change through emissions reduction are meeting with limited success, policymakers are increasingly looking to reduce vulnerability by promoting adaptation (11), and many climate change vulnerability frameworks include adaptation (11, 12).

Vulnerability analyses should consider the effect of social and political conditions on vulnerability (10), and have been used to address the potential effects of climate change on the health of marginalised groups (13). Extensive literature has shown that policies that do not consider local knowledge, values and lifestyles cause conflict and hardship (14–16), and Turner et al. (10) recommend including stakeholders in vulnerability analyses, namely by identifying outcomes to be avoided and their drivers. This project uses a participatory vulnerability analysis that starts with community identification of harmful outcomes, as proposed by Turner et al. (10), and similar to that used by Berrang-Ford et al. (13).

## Traditional knowledge

Considerable research demonstrates the value of the traditional knowledge held by indigenous resource users (17, 18), as observations made over generations can be considered large samples with greater spatial and temporal continuity than the observations recorded by many Western science studies (19). Indigenous users often observe features of and changes in local environments that go unnoticed by resource managers (20), and numerous scholars argue that traditional knowledge can improve environmental policymaking (21). While the Western science on ice seals, walruses and sea ice is relatively new and has many gaps, indigenous hunters have extensive observations of both marine mammals and on-going changes in ice and weather conditions (3, 22). As such, a participatory vulnerability analysis not only ensures that local livelihoods and values are considered appropriately, it can generate important new information on the environmental drivers of vulnerability.

## Study area

**Fig. 1:**
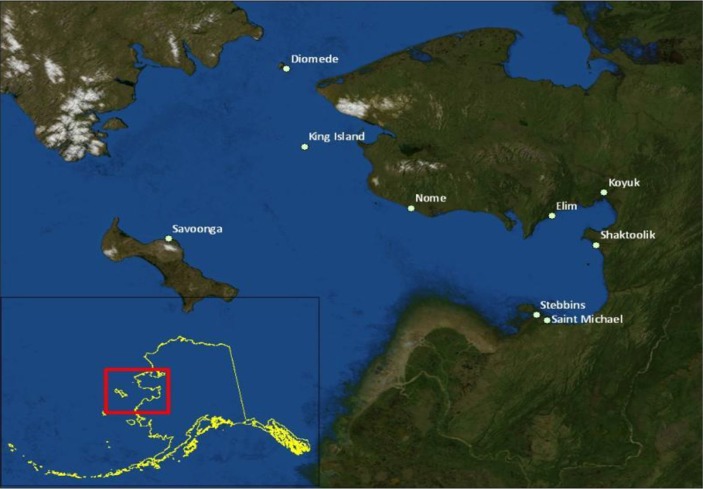
Study area.

Kawerak, Inc. is the Alaska Native non-profit corporation for the Bering Strait region and represents tribes in the Norton Sound and Bering Strait areas. 9 communities participated in this project: Nome, King Island, Diomede, Savoonga, Elim, Koyuk, Shaktoolik, Stebbins and St. Michael (Fig. 1).

## Methods

This research is part of a larger project on traditional knowledge of ice seals and walruses conducted by Kawerak's Social Science Program in collaboration with the Eskimo Walrus Commission, the Ice Seal Committee and Oceana. All research was conducted to the standards recommended by the Interagency Arctic Research Policy Committee (23), which includes informed consent, tribal government approval, community involvement in research development, local assistants, honorariums for participants, data security and community review of research results.

### Participatory research design

Public meetings and meetings with tribal governments were held in participating communities to gather input on community research priorities. Potential ice seal and walrus research topics were presented for consideration, and community input was written down in note form. Draft interview topics were also presented to the Ice Seal Committee and Eskimo Walrus Commission for feedback. Meeting notes were typed up and coded in Atlas.ti. The coded information was used to design the question sets for interviews and focus groups, which were then tested on Kawerak employees and an elder from Diomede.

### Data collection, processing and analysis

Semi-structured interviews and focus groups were conducted with a purposive sample (n =82) of local expert hunters and elders (24) identified by tribal governments and other expert hunters in the 9 participating communities. Interview topics relevant to this analysis included the importance of seal and walrus hunting, animal health and food safety, concerns about and perceived threats to seal and walrus hunting, and recommendations for addressing these threats. Interview content varied according to hunter, and interviewers added follow-up questions as needed.

Interviews and focus groups were digitally recorded, transcribed and coded in either Atlas.ti or in Word. Both deductive and inductive codes were used (25), with deductive codes primarily generated during the participatory research design process.

### Importance of Seal and Walrus Hunting

Quotes coded under “importance” were summarised and organised into the inductively generated categories of self-determination, health/food security, cultural preference and lifestyle/identity.

### Vulnerability analysis

Quotes coded under “concerns/recommendations” were used to identify undesirable outcomes and potential preventative measures. Information relevant to identified outcomes was extracted and summarised from other relevant codes (disturbance, environmental changes, safety and species specific codes for marine mammal health and behavior) and categorised using the standard climate change vulnerability analysis categories of exposure, sensitivity and adaptive capacity (12, 26). For the purposes of this analysis, exposure is the direct cause of the undesired outcome, sensitivity is the reason the exposure causes harm and adaptations are methods to prevent either the exposure or the capacity of the exposure to do harm.

### Food safety

Participants were asked: (a) if they had ever seen sick or abnormal marine mammals and (b) to describe how they determined if marine mammals were safe to eat. This information was coded by species under the sub-code “population size and health”. For this paper, information was aggregated from all species and then summarised and organised according to the inductively generated categories normal/abnormal and edible/not edible.

## Results

### Importance

Participants described a variety of reasons that marine mammal harvests were important to them, their families and communities (Table I). Major themes that arose included the right to self-determination, the health benefits of and local preferences for marine mammal foods, and the role of marine mammal harvesting in food security, personal identity, cultural practices and the transmission of cultural knowledge.

**Table I:** Importance of marine mammal harvests to participants

Self-determination
People should have the right to eat traditional cultural foods, to pursue traditional cultural activities and livelihoods, and to pass traditions on to the next generation.
Health and food security
Non-native foods are more likely to cause diabetes and heart disease Marine mammal oils are used to preserve other native foods. Marine mammal foods are portable and keep hunters warm and full when hunting. Stores do not always have food available in isolated villages. The rural cash economy is unstable; people will not always have money to buy food from the store.
Cultural preference
Native foods are preferred foods, and seal oil is an essential condiment. Some people cannot eat food without seal oil. *They prefer seal oil over mayonnaise or ketchup*. *We grew up using that seal oil … we have to have it*. *It's food that I grew up with. And when I don't eat it, I always tell my wife, ‘I'm starving’*. *That's our beef. Our beef from the ocean*.
Lifestyle/identity
Hunting is a very important part of identity. Preparing, sharing, and consuming native foods are important cultural activities. Children learn cultural traditions by participating in marine mammal harvesting and preparation. Marine mammal parts are needed to make items such as drums and clothes for cultural activities. Handicrafts from marine mammals provide income.

Non-italicised phrases are paraphrased from quotes, italicised phrases are direct quotes.

### Vulnerability

Marine mammal hunters discussed a number of potential undesirable outcomes that could be caused by climate change and resultant industrial development. These outcomes were contextualised with interview data categorised as exposure, sensitivity and preferred adaptation (Table II). Preferred adaptations fell into 4 main categories: traditional knowledge documentation, policy advocacy, technology and research (Table III). Most proposed adaptations recommended policy changes or traditional knowledge documentation.

**Table II:** Participants' concerns and recommendations organised by vulnerability analysis categories

Exposure	Sensitivity	Outcome	Adaptation
Disturbance from increased shipping	Marine mammals have excellent hearing and communicate through sound	Marine mammals: May displace	Document traditional knowledge of habitat areas
	Marine mammals equate certain noises with danger	Hunters: Must go farther to hunt, or may not be able to find marine mammals	Designate shipping lanes to avoid important areas
			Regulate noise pollution
Deteriorating ice conditions	Marine mammals rest, pup, and calve on ice	Marine mammals: New diseases, poor condition	Document traditional methods for determining food safety
		Hunters: Concerns about food safety and access to healthy seals	Submit samples for testing, support research on marine mammal diseases
Deteriorating ice and weather conditions	Hunters in small boats are at risk from dangerous ice and weather conditions	Hunters travel farther in worse conditions and are more at risk of hunting accidents	Document traditional knowledge of safe harvesting practices
	Hunters must travel farther when ice conditions are poor		Use new safety technologies
Pollution from increased shipping and development	Marine mammals accumulate toxins	High levels of toxins in marine mammals and humans	Regulate pollution from shipping
	Humans eat marine mammal oils and organs, which concentrate toxins		Send samples for testing, support research on marine mammal contaminant load
Bottom-trawling follows fish into northern Bering Sea	Ocean bottom recovers slowly	Marine mammals: population reduction or displacement	Oppose bottom trawling
	Walrus and bearded seals are benthic feeders	Hunters: food security decreases through reduced access	Document important feeding areas
Ice seals and/or walruses listed as threatened or endangered under the Endangered Species Act	Lack of local influence on federal policies	Hunters: Concern that regulations may make traditional uses difficult	Advocate for greater local representation in federal management
	Groups that do not understand local traditions exert control over federal policies		Advocate for more power for co-management groups
			Document marine mammal adaptive capacity
			Document traditional management strategies

Phrases are summaries of participant responses.

**Table III:** Proposed adaptations by category

Traditional knowledge documentation	Policy advocacy	Technology	Research
Document traditional knowledge of habitat areas	Designate shipping lanes to avoid important areas	Use new safety technologies such as weather forecasts and GPS	Support research on marine mammal diseases and contaminant load by submitting samples for testing
Document traditional methods for determining food safety	Regulate noise pollution (speed and ship design)		
Document traditional knowledge of safe harvesting practices	Regulate pollution from shipping		
Document important marine mammal feeding areas	Oppose bottom trawling		
Document marine mammal adaptive capacity	Advocate for greater local representation in federal management		
Document traditional management strategies	Advocate for more power for co-management groups		

Phrases are summaries of participant responses. Columns are independent and information is not comparable across rows.

### Traditional methods for determining if marine mammal food is safe to eat

Hunters explained that when they harvest a seal or walrus, they know what is normal and what is not normal. As one hunter explained, “we're not gonna eat something that doesn't look normal, we know what normal is”. Marine mammals are examined for parasites, deformed organs, and, as another hunter elaborated, “the fatness of the blubber and the colour of the skin”. In Tables IV and V, observed conditions are categorised as normal or abnormal and edible or inedible. Note that all abnormal conditions are considered inedible.

**Table IV:** Normal conditions hunters have found in seals and walruses

Normal: edible	Normal: not edible
Yellow blubber Scars (especially bearded seals) Some hair loss Tapeworms in bearded seal stomachs (but not in meat) Rocks in stomachs of otherwise healthy walruses, seals, or bearded seals A little skinny but otherwise normal	Seal-eating walrus Rutting (gasoline) seals (unpalatable to most but not unsafe to eat)

**Table V:** Abnormal conditions hunters have found in seals and walruses

Not normal, not edible
Rotten smell (even when alive) Very skinny Bloated Lesions/sores Lethargic/approachable Sores on tusks Lumps inside and outside of body Puss around eyes Discoloration of skin, meat, liver or tissues Liver flukes Worms in heart Infected liver and kidneys Black lesions on kidneys

## Discussion

Hunter responses indicated that preferred adaptations fell into 2 main categories: (a) policy advocacy informed by traditional knowledge and local use patterns and (b) traditional knowledge documentation for both policy advocacy and local use. Participants universally expressed a desire to preserve marine mammal harvesting traditions, and no proposed adaptations involved switching to other food sources. Although some researchers have suggested that communities need access to more locally applicable research in order to respond to climate change (27), participants in this study were generally more interested in creating precautionary environmental policies based on existing traditional knowledge than in receiving new information from scientific research. Participants did note that they would like more information about marine mammal contaminants and emerging marine mammal sicknesses. Most participants were also uninterested in having data about their own use and knowledge collected for decision-makers from other places. While this study does not contradict Moser and Ekstrom's (28) claim that limitations in data/information availability and usability are common barriers to climate change adaptation, it does indicate that in some cases, traditional or local knowledge may be more relevant information than scientific research.

Overall, project participants wanted the political power to generate local solutions using traditional knowledge and to regulate industrial development in their traditional use areas. As a participant from Saint Lawrence Island noted:

The more I read about the way the U.S. and other countries are planning to use this northern routing, it's as if we don't exist there. They're just talking about how easy it is going to be for them to go from one country to [another] country using this polar sea lane. And that is good for a lot of countries out there. But we need to get the respect here on the island, that there are people here that live off the Bering Sea. And that's the respect we [want to] see from the world … otherwise people here will starve.

Participant concerns about influencing policy are consistent with other climate change adaptation research indicating that limitations of authority and influence can act as a barrier to climate change adaptation (28) and that institutional linkages that connect communities with policymakers at higher levels can increase the resilience of a system (29). This indicates that a strategy to promote rural Alaskan adaptation to climate change would be to better incorporate traditional knowledge and local values into decision-making processes. Participant interest in documenting traditional knowledge for local use also indicates that funding agencies could support climate change adaptation by awarding more grants for tribal research that advances local, rather than academic, use of traditional knowledge.

## Conclusion

This research discusses the connection between human health and ocean health. Indigenous marine mammal hunters of the Bering Strait region described the cultural and nutritional importance of seal and walrus harvests. For many in the region, participating in marine mammal hunting and eating traditional foods are essential components of a healthy lifestyle. Traditional use of marine mammals may be threatened by climatically-induced deterioration of sea ice and resultant industrial development. Overall, participants stated that greater local participation in environmental decision-making, as well as local efforts to document relevant traditional knowledge, would help communities decrease their vulnerability to climatic changes. Specifically, participants described how a variety of environmental regulations on ship speed, noise, and discharge, as well as prohibitions on industrial fishing, would help mitigate harmful effects on marine mammals, and thus on the humans who depend on those species. Additionally, participants expressed an interest in documenting traditional marine mammal management strategies, and traditional knowledge of marine safety, marine mammal behavior, and habitat.

## References

[1] ACIA (2004). Impacts of a warming Arctic: Arctic climate impact assessment. http://www.acia.uaf.edu.

[2] Ahmasuk A, Trigg E, Magdanz J, Robbins B (2008). A comprehensive subsistence use study of the Bering Strait region.

[3] Oozeva C, Noongwook C, Noongwook G, Alowa C, Krupnik I, Krupnik I, Huntington H, Koonooka C, Noongwook G (2004). Watching ice and weather our way.

[4] Steele M, Ermold W, Zhang JL (2008). Arctic Ocean surface warming trends over the past 100 years. Geophys Res Lett.

[5] Kwok R, Cunningham GF, Wensnahan M, Rigor I, Zwally HJ, Yi D (2009). Thinning and volume loss of the Arctic Ocean sea ice cover: 2003–2008. J Geophys Res.

[6] Krupnik I, Aporta C, Gearheard S, Laidler G, Kielsen Holm L (2010). SIKU – knowing our ice – documenting Inuit sea ice knowledge and use.

[7] Fay FH (1982). Ecology and biology of the Pacific Walrus *Odobenus rosmarus divergens Illiger*.

[8] Kelly BP, Lentfer JW (1988). Bearded Seal *Eriganthus barbathus*. Selected marine mammals of Alaska: species accounts with research and management recommendations.

[9] Raymond-Yakoubian J, Carothers C, Criddle K, Chambers C, Cullenberg P, Fall J, Himes-Cornell A (2012). Participation and resistance: tribal involvement in Bering Sea fisheries management and policy. Fishing people of the North: cultures, economies, and management responding to change.

[10] Turner BL, Kaskerson RE, Matson PA, McCarthy JJ, Corell RW, Christensen L (2003). A framework for vulnerability analysis in sustainability science. Proc Natl Acad Sci.

[11] Ford JD, Smit B (2004). A framework for assessing the vulnerability of communities in the Canadian Arctic to risks associated with climate change. Arctic.

[12] Parry ML, Canziani OF, Palutikof JP, van der Linden PJ, Hanson CE, IPCC (2007). Climate change 2007: impacts, adaptation and vulnerability. Contribution of Working Group II to the Fourth Assessment Report of the Intergovernmental Panel on Climate Change.

[13] Berrang-Ford L, Dingle K, Ford JD, Lee C, Lwasa S, Namanya DB (2012). Vulnerability of indigenous health to climate change: a case study of Uganda's Batwa Pygmies. Soc Sci Med.

[14] Ostrom E (2005). Understanding institutional diversity.

[15] Berkes F (2007). Community-based conservation in a globalized world. Proc Nat Acad Sci.

[16] Reynolds JF, Smith DM, Lambin EF, Turner BL, Mortimore M, Batterbury SP (2007). Global desertification: building a science for dryland development. Science.

[17] Acheson JM, Wilson JA, Steneck RS, Berkes F, Folke C (1998). Managing chaotic fisheries. Linking social and ecological systems: management practices and social mechanisms for building resilience.

[18] Holling CS, Berkes F, Folke C, Berkes F, Folke C (1998). Science, sustainability, and resource management. Linking social and ecological systems: management practices and social mechanisms for building resilience.

[19] Moller H, Berkes F, Lyver PO, Kislalioglu M (2004). Combining science and traditional ecological knowledge: monitoring populations for co-management. Ecol Soc.

[20] Ray L, Kolden CA, Chapin FS (2012). A case for developing place-based fire management strategies from traditional ecological knowledge. Ecol Soc.

[21] Failing L, Gregory R, Harstone M (2007). Integrating science and local knowledge in environmental risk management: a decision-focused approach. Ecol Econ.

[22] Krupnik I, Ray GC (2007). Pacific walruses, indigenous hunters and climate change: bridging scientific and indigenous knowledge. Deep Sea Res. PT II.

[23] NSF (1990). Principles for the conduct of research in the Arctic. http://www.nsf.gov/od/opp/arctic/conduct.jsp.

[24] Chalmers N, Fabricius C (2007). Expert and generalist local knowledge about land-cover change on South Africa's Wild Coast: can local ecological knowledge add value to science?. Ecol Soc.

[25] Marshall C, Rossman GB (1995). Designing qualitative research.

[26] Ford JD, Berrang-Ford L, King M, Furgal C (2010). Vulnerability of Aboriginal health systems in Canada to climate change. Global Environ Change.

[27] Wilbanks TJ, Kates RW (1999). Global change in local places: how scale matters. Climatic Change.

[28] Moser SC, Ekstrom JA (2010). A framework to diagnose barriers to climate change adaptation. Proc Nat Acad Sci.

[29] Berkes F, Jolly D (2001). Adapting to climate change: social-ecological resilience in a Canadian western Arctic community. Conserv Ecol.

